# Public health round-up

**DOI:** 10.2471/BLT.23.010523

**Published:** 2023-05-01

**New mosquito nets needed**

A mother and baby sleep under an insecticide-treated net (ITN) in the village of Moujia, in the Tahoua region of Niger. Since 2005, over two billion ITNs have been distributed worldwide, significantly reducing malaria transmission. However, in the face of increasing insecticide-resistant mosquitoes, new nets are needed. On 14 March, the World Health Organization (WHO) published recommendations for two new classes of dual-ingredient ITNs with a view to tackling the problem.

**Figure:**
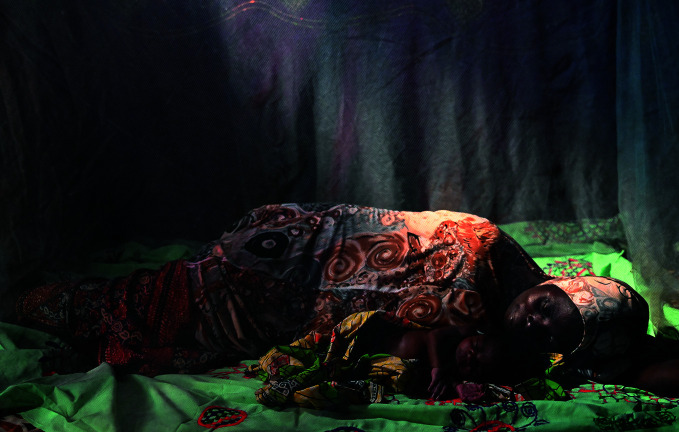
UNICEF Frank Dejongh

## Dengue and chikungunya in the Americas

The increased incidence and geographical spread of arboviral diseases in the Region of the Americas is causing concern. A total of 3.1 million cases were reported in 2022.

An estimated 2.8 million people were infected with dengue virus, 1290 of whom died. This represents a two-fold increase in cases and an almost three-fold increase in deaths compared with 2021, when 1.3 million people were reported to have been infected, 437 of whom died. As of 4 March, just over 342 000 cases, and 86 deaths had been reported this year.

Chikungunya cases are also rising, with some 274 000 people reported to have been infected in 2022, 87 of whom died. This represents a two-fold increase in cases and seven-fold increase in deaths compared with 2021.

As of 4 March, a total of just over 113 000 cases of chikungunya had been reported this year, including 51 deaths – a four-fold increase in cases and deaths compared with the same period in 2022. The geographical expansion of chikungunya cases is also a concern, as are cases of acute meningoencephalitis that are being attributed to chikungunya in Paraguay.

Prevailing high temperatures and humidity, favouring the proliferation of mosquitoes, are expected to drive higher transmission of arboviruses in the coming months.

The World Health Organization (WHO) is supporting Member States’ preparedness and outbreak response efforts in the region.

http://bit.ly/3KJO570

## Marburg firsts in Africa

The Ministry of Health of the United Republic of Tanzania declared its first outbreak of Marburg virus disease. As of 22 March, a total of eight people, two of whom health workers, were reported to have been infected in the Kagera region, five of whom had died.

Rapid response teams were deployed to investigate and to initiate contact tracing among other measures.

In light of cross-border movements between Kagera region residents and bordering countries including Uganda in the north and Rwanda and Burundi in the west, WHO assessed the risk of spread to be very high at the national level, and high at the subregional level. The risk of global spread was assessed to be low.

Public health authorities in Equatorial Guinea are also dealing with their first outbreak of Marburg, which was first declared in February. As of 22 March, 29 people were reported to have been infected (nine laboratory confirmed, 20 probable). All but two of the people had died.

As of 21 March, there was no evidence of an epidemiological link between the outbreaks in the two countries.

http://bit.ly/3nPIWRO

http://bit.ly/3m7cs54

## Multi-country cholera outbreaks

The global cholera situation continued to deteriorate, with a total of 24 countries reporting cases as of 20 March. The simultaneous progression of multiple outbreaks, the impact of which is compounded in countries facing complex humanitarian crises with fragile health systems and aggravated by climate change, poses challenges to outbreak response and favours further spread.

Based on the current situation, including the increasing number of outbreaks and their geographic expansion, as well as a lack of vaccines and other resources, WHO assesses the risk at the global level as very high.

https://bit.ly/3KfhSmR

## Avian influenza in Chile

The Chilean Ministry of Health notified WHO of a laboratory-confirmed human infection with avian influenza A(H5) virus. The patient is a 53-year-old male from the Antofagasta region in northern Chile.

This is the first human infection with avian influenza A(H5) virus reported in Chile and the third reported in the Region of the Americas to date. As of 8 April, no further human cases had been identified, although one of the health workers who had contact with the man and had developed respiratory symptoms, was being examined. An outbreak investigation had been initiated.

Unprecedented outbreaks of highly pathogenic avian influenza variants in animals had been reported from Chile in the months prior to the case, including viruses detected among backyard poultry, farm poultry, wild birds, and sea mammals.

http://bit.ly/3GzvyI7

## WHO 75^th^ anniversary

WHO celebrated its 75th anniversary on 7 April, marking the occasion with a call for a renewed drive towards health equity and global cooperation.

“The history of WHO demonstrates what is possible when nations come together for a common purpose,” said Tedros Adhanom Ghebreyesus, WHO Director-General. “We have much to be proud of, but much work to do to realize our founding vision of the highest attainable standard of health for all people. We continue to face vast inequities in access to health services, major gaps in the world’s defences against health emergencies, and threats from health-harming products and the climate crisis.”

To meet these challenges, WHO is urging countries to take urgent action to protect, support and expand the health workforce to avert a projected shortage of 10 million health workers by 2030 (down from an estimated 15 million currently), primarily in low- and middle-income countries.

http://bit.ly/40G3VoJ

## Stepping up efforts to tackle tuberculosis

WHO released details of an expanded WHO Director-General’s flagship initiative on tuberculosis (TB). Released 23 March (World TB day) and covering the period 2023-2027, the expanded initiative builds on the progress achieved and lessons learned since 2018 when the initiative was launched, and focuses on ensuring universal access to prevention, care and the latest tools and technologies to combat tuberculosis as part of the drive towards universal health coverage.

http://bit.ly/3ZVusgw

## New nets to tackle mosquitoes

WHO published recommendations for two new classes of dual-ingredient, insecticide-treated nets (ITNs) with a view to tackling insecticide-resistant mosquitoes.

Since 2005, over two billion ITNs have been distributed worldwide, all treated with only one insecticide class – pyrethroids. The ITNs have contributed significantly to the progress made in reducing malaria cases, however as mosquitoes in many areas are now resistant to pyrethroids, nets treated with other active ingredients are needed.

In 2017, WHO started to recommend a new type of ITN that combines pyrethroids with piperonyl-butoxide (PBO), a chemical that enhances the potency of pyrethroids against resistant mosquitoes.

Now the Organization is recommending two new classes of dual-ingredient ITNs with different modes of action that enhance the nets’ insecticidal effect and disrupt mosquito growth and reproduction.

The recommendations were published in the *WHO guidelines for malaria* on 14 March, along with new guidance to support national malaria programme decision-making on which nets to prioritize.

http://bit.ly/43kEoTJ

**Cover photo**

A vaccinator and community health worker cross a dry riverbed as they walk to a village in Lehele, Kenya to administer oral cholera vaccines, February 2023.

**Figure:**
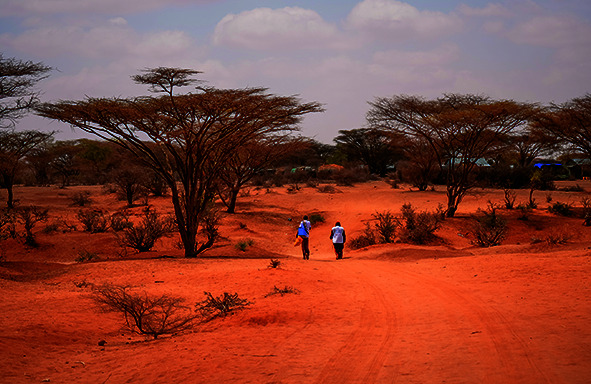
WHO / Billy Miaron

## Draft pandemic accord

WHO Member States agreed on modalities and a schedule for negotiations on a global accord on pandemic prevention, preparedness and response, with a view to presenting a draft accord for approval by the World Health Assembly in May 2024.

Ending 6 April, discussions took place during the fifth meeting of the Intergovernmental Negotiating Body (INB) which is comprised of the 194 Member States.

http://bit.ly/3Go0s6a

## Boosting motorcycle helmet use

WHO published a new manual designed to support increased use of safe motorcycle helmets. Launched on 5 April, *Helmets: a road safety manual for decision-makers and practitioners*, presents new guidance on the laws, regulations and actions needed to advance the safe helmets agenda, underlining the fact that, despite the rapid increase in the number of motorcycles, especially in low-and middle-income countries, helmet use in those countries remains low.

The manual was launched at the Bloomberg Philanthropies Initiative for Global Road Safety Regional Meeting for Asia in Mumbai, India. ‘Urgent action is needed to stave off a rapid rise in deaths and injuries in the coming years,’ said Matts-Ake Belin, global lead for the United Nations Decade of Action for Road Safety 2021–2030 at WHO.

http://bit.ly/3zCHBk4

**Looking ahead**

4–5 May 2023. 1^st^ Global Joint Summit of Human and Veterinary Medicines Regulatory Authorities to Preserve Antimicrobials. Geneva, Switzerland. http://bit.ly/3L5Pllr

8–11 May 2023. International Maternal Newborn Health Conference. Cape Town, South Africa. http://bit.ly/438qu7l

21-30 May 2023. Seventy-sixth World Health Assembly. Geneva, Switzerland. https://bit.ly/3IK55GL

